# Fever and Ague Prevented

**Published:** 1869-10

**Authors:** 


### FEVER AND AGUE PREVENTED.

The sun-flower, planted around a house in a locality where intermittents prevailed, was observed, before the war, by a gentleman connected with the United States Government to protect the whole family from that troublesome sickness. The subject has been presented by M. Martin to the French Society for the Promotion of the General Health, and the

Italian Sanitary Officers have taken measures to put the suggestion in operation in the most sickly localities. While the editor was a medical student, it was stated by Professor Cook, that, in all marshy countries, a thick hedge of vegetable growth, from ten to fifteen feet high, should be had, some forty or fifty feet from the dwelling, especially in the direction of stagnant water, mill-ponds or marshy places where there was growing and decaying vegetation. His theory was this, that miasm was generated in all such localities; that in the morning and evening, about sunrise and sunset, this miasm was concentrated on the surface of the earth, extending eight or ten feet upwards, allowing it to be breathed into the lungs, taken in upon an empty stomach, engendering all forms of intermittents and other more dangerous fevers, diarrheas, dysentery and cholera; but that a thick, living, vegetable growth had been known by him for many years, from actual observation, to arrest the flow of miasm towards the house and save the inmates from disease.

The object in making this statement at such length is to show that the absorption of miasm was not peculiar to the sunflower, but that it was living vegetation; and, as there are other forms more luxuriant and more dense than the sunflower, such growths are best; and that these facts were published in 1828, in John Esten Cook's Therapeutics. It was further suggested that the growth should not be nearer the house, because it would keep it damp, and, for the same reason, that although it looked well to see a country house embowered and almost hid by trees, it was not healthful; that the sunshine should come upon every part and side of a dwelling in summer and winter, otherwise disease would prevail; that the sunlight was everywhere a great promoter of health.

